# Retraction: Abdalla, S., et al. A Bio Polymeric Adhesive Produced by Photo Cross-Linkable Technique. *Polymers* 2016, *8*, 292, doi:10.3390/polym8080292 and Abdalla, S., et al. Controlled Light Cross-Linking Technique to Prepare Healable Materials. *Polymers* 2017, *9*, 241, doi:10.3390/polym9060241

**DOI:** 10.3390/polym9080382

**Published:** 2017-08-21

**Authors:** Polymers Editorial Office

**Affiliations:** MDPI AG, St. Alban-Anlage 66, 4052 Basel, Switzerland

These two articles [1,2] published in *Polymers* will be marked as retracted. It has come to our attention that more than half of the figures in the published paper [1] and all schemes and figures in the published paper [2] are copied from two previous publications [3,4]. We consider this to be a serious breach of publication ethics.

We very much regret that the plagiarism of these figures was not detected prior to publication. We would like to offer our apologies to readers of *Polymers* and wish to thank the readers who brought it to our attention. *Polymers* is a member of the Committee on Publication Ethics (COPE) and strives to uphold the highest ethical standards. We are committed to taking appropriate action when such cases are reported.
